# *BRCA* Testing Dichotomy in Saudi Arabia

**DOI:** 10.1200/JGO.18.00188

**Published:** 2019-02-01

**Authors:** Atlal Abusanad

**Affiliations:** ^1^ King Abdulaziz University, Jeddah, Saudi Arabia

*BRCA* 1 and 2 were recognized as predisposing genes for hereditary breast and ovarian cancer in 1990 and 1994, respectively.^1,2^ Since then, the *BRCA* tale has evolved from being a predictor for increased risk of certain malignancies into a therapeutic biomarker, predicting response to agents that interfere with DNA damage repair, such as poly (ADP-ribose) polymerase inhibitors and potentially platinum-based chemotherapies.^3,4^ Recent approval of poly (ADP-ribose) polymerase inhibitors meant increasing value for *BRCA* testing in patients’ care.^5-7^ Despite the well-recognized indications for *BRCA* testing, the test is offered sparsely in certain areas of the world, including Saudi Arabia.^8^ Several barriers to testing exist, including the scarcity of testing facilities and the logistic difficulties linked to sending biologic samples abroad.^9^ Additional challenges include the lack of well-established cancer genetic clinics and related support staff and the refusal of patients and/or their families to undergo testing^10^.

Testing for mutations associated with other cancers is typically faced with less hesitancy from the patient side, despite having similar benefits to *BRCA* testing in terms of the potential to guide treatment choices. In patients with non–small-cell lung cancer, for instance, epidermal growth factor receptor mutation, a somatic mutation acquired during carcinogenesis, is not transmissible to offspring^11,12^; thus, it carries no consequences for other family members and is devoid of social stigma. *BRCA* mutations, however, are germline mutations transmitted in autosomal dominant fashion. Each one of the affected individual’s offspring has a 50% chance of acquiring the mutated allele, whereas the second allele mutation might occur during the lifetime with resultant carcinogenesis. Consequently, the perception of *BRCA* mutations and their association with hereditary cancer syndrome can be highly stigmatizing.^13^ In Saudi Arabia, the impact of a *BRCA* diagnosis may result in a social burden. Research shows that the potential negative ramifications on other family members, especially young daughters or sisters, make *BRCA* testing less desirable to patients.^11^ Our experience tells us that many patients in Saudi Arabia prefer to keep silent about a cancer diagnosis; therefore, they are expected to feel uneasy about testing for a condition that increases the risk of breast cancer by six- to eight-fold and ovarian cancer by four- to six-fold.^14^ Needless to say, the paucity of trained staff to address and manage the follow-ups, surveillance, and necessary risk reduction interventions for any diagnosed *BRCA* family further worsens the situation. Saudi oncologists certainly have to offer such *BRCA* testing to candidates; however, how they are supported by allied services and prepared to address the consequences of testing presents challenges.

We are faced with the unique situation of having a therapeutic biomarker with inheritance potential not comparable to currently used biomarkers. The *BRCA* dichotomy is illustrated in the situation where a patient who may benefit from *BRCA* testing declines it because of her concerns about the consequences of the results. With *BRCA* testing becoming an integral part of patient care, the health care system and society have to be prepared to deal openly with such circumstances at the psychological, social, and medical levels.

## Data Availability

The following represents disclosure information provided by authors of this manuscript. All relationships are considered compensated. Relationships are self-held unless noted. I = Immediate Family Member, Inst = My Institution. Relationships may not relate to the subject matter of this manuscript. For more information about ASCO's conflict of interest policy, please refer to www.asco.org/rwc or ascopubs.org/jco/site/ifc. No potential conflicts of interest were reported.
